# Advancing Aging Equity in Dementia: Structural and Psychosocial Factors, Community Engagement and Inclusion

**DOI:** 10.1093/geroni/igaf122.1094

**Published:** 2025-12-31

**Authors:** Crystal Glover

**Affiliations:** University of California, Irvine, Irvine, California, United States

## Abstract

Disparities in aging and dementia stem from deeply embedded structural and psychosocial determinants of health. Systemic inequities in healthcare access, socioeconomic status, and education create significant barriers to early diagnosis, treatment, and long-term support for minoritized older adults. At the same time, psychosocial determinants—including trust in research, cultural beliefs, and caregiving burdens—further shape the experiences of aging and dementia within historically underrepresented communities. Ensuring progress in aging equity requires sustained, community-driven strategies that foster inclusion, build trust, and challenge existing barriers to participation. This presentation examines how structural and psychosocial factors contribute to disparities in dementia research and care while advancing innovative, evidence-based approaches to expand equitable participation. Recent findings reinforce the critical need for participatory research models that actively engage diverse populations and address longstanding distrust. The ENGAGED model exemplifies a framework for transforming outreach and recruitment by integrating bidirectional knowledge exchange, culturally responsive engagement, and sustained community partnerships. By embedding equity at the core of both research and practice, a scalable and empirically grounded blueprint emerges—one that sustains progress, innovates inclusion, and ensures that all older adults, regardless of race, ethnicity, or socioeconomic background, are equitably represented in aging and dementia research.

